# Larger contactor area increases low-frequency vibratory sensitivity in hairy skin

**DOI:** 10.7717/peerj.8479

**Published:** 2020-02-03

**Authors:** Daniel Schmidt, Guenther Schlee, Andresa M.C. Germano, Thomas L. Milani

**Affiliations:** 1 Department of Human Locomotion, Faculty of Behavioral and Social Sciences, Institute of Human Movement Science and Health, Chemnitz University of Technology, Chemnitz, Germany; 2 Biophysics and Human Performance Lab, W.L. Gore and Associates, Putzbrunn, Germany

**Keywords:** Vibration perception thresholds, Spatial summation, VPTs, Cutaneous sensitivity, Contactor size, Sensitivity

## Abstract

**Background:**

In research, assessing vibratory cutaneous sensitivity is an important research branch to quantify various diseases or to develop devices for pattern recognition. The measured vibration perception thresholds (VPTs), however, are subjective and usually result in a large data variability. This might induce difficulties to detect differences, for example, when comparing different anatomical locations. Hence, a higher ability to detect changes is desirable. Another feature of VPTs is spatial summation, but in the literature it is controversially discussed whether or not this phenomenon is also present in the lower frequency range. For these reasons, the present study aimed to investigate whether an enlarged matrix contactor area (measured at the hairy skin) induces improvements in subjective sensitivity using high and low frequencies, and whether a large contactor area is better able to identify changes of VPTs than a small contactor area of a single contactor. For each frequency, we hypothesized an increased sensitivity for the matrix compared to the single contactor. We also hypothesized that changes can be better-detected between the anatomical locations when using the matrix than the single contactor.

**Methods:**

Twenty healthy and young participants voluntarily took part in this study. Three anatomical locations at the torso were measured at the middle aspect of the lower back, middle lateral aspect of the upper arm, and the region just below the armpit. At each location, two frequencies (30, 200 Hz) and two contactor conditions (single contactor: 0.48 cm^2^ , contactor matrix: 9 × 0.48 cm^2^ = 4.32 cm^2^) were tested in a randomized order.

**Results:**

Supporting our hypothesis, we found that improved cutaneous sensitivity after increasing the contactor size occurs not only at high, but also at low frequencies at all anatomical locations. Large contactor sizes resulted in higher sensitivity and in a superior ability to detect changes. The superior behavior of the matrix to exhibit a lower variability could not always be proven. This work may be relevant for future studies aiming to identify changes of VPTs in various patient groups, for example.

## Introduction

Measuring cutaneous sensitivity is a widely implemented branch of research. Human skin, including its receptors and the subsequent afferent nerve tissues, allows us to perceive various external stimuli (such as pressure, temperature, or vibration). Based on this, the central nervous system generates appropriate motor responses, for example, after touching a pointy or hot item. One particular feature of cutaneous sensitivity is its ability to detect vibrations. This is achieved by mechanoreceptors located within the skin. For example, Meissner corpuscles, which are present in glabrous skin, are most sensitive in the lower frequency range (Strzalkowski, Ali & Bent, 2017), whilst Vater–Pacini corpuscles (present in both glabrous and hairy skin) are known to be highly sensitive to high-frequency vibrations (around 200 Hz (Birznieks et al., 2019)).

Vibration stimuli are used, for example, to develop vibrotactile supportive devices for pattern recognition (Jones, Lockyer & Piateski, 2006) for disabled people, such as the blind. For instance, various properties of obstacles on the ground (dimensions, distance, direction, etc.) are coded into vibrations and then transmitted onto the skin of various torso regions using vibrotactile vests. Another important application of vibrotactile stimuli is to quantify various neurological diseases. This is mainly achieved by assessing vibration perception thresholds (VPTs). VPTs correspond to the smallest perceivable amplitude of a vibration stimulus at a given frequency. Many neurological diseases, for example, diabetic neuropathy, negatively influence VPTs: The amplitude of the stimulus necessary to elicit the perception of the vibration is increased (Garrow & Boulton, 2006). In other words, sensitivity is decreased. VPTs have even been used to predict the presence of neuropathic foot ulceration (Young et al., 1994), or to examine the function and performance of the human foot in anthropological studies (Holowka et al., 2019).

Of course, assessing VPTs is a subjective method, as the subject indicates whether the amplitudes of different vibratory stimuli were perceived or not. It was discovered as early as 1954 that VPTs are individual, even when testing the same anatomical location in different subjects (Wilska, 1954). Such data typically exhibit large variability, which makes inferential statistical comparisons between populations (e.g., healthy vs. diseased) difficult. This is also true when comparing various anatomical locations, or when doing longitudinal comparisons (e.g., test–retest scenarios). Hence, the potential variability of VPTs may constitute a limitation regarding their ability to detect (clinically relevant) differences. In the past, another subjective method to assess cutaneous mechanical sensitivity (vertical pressure stimuli) demonstrated the inability to differentiate between healthy and diseased subjects (Pagel, Kaul & Dryden, 2002). This highlights the problematic character of subjective assessment methods. Hence, reducing data variability seems desirable.

It is also known that spatial summation occurs under certain circumstances with cutaneous vibratory stimuli. Spatial summation may be explained as an increased likelihood to detect a (vibratory) stimulus as the contactor size is enlarged. Bolanowski, Gescheider & Verrillo (1994) define this term as an increased sensitivity induced by a greater number of receptors being activated due to a larger stimulus area. However, the increased sensitivity is not determined by the neural density per se, but rather is due to the increased number of sensory afferent units activated by the stimulus (Verrillo & Chamberlain, 1972). This phenomenon is usually only associated with the Pacinian channel as one information-processing channel.

For glabrous skin, it is proposed that there are four separate information-processing channels to mediate mechanical stimuli, mainly induced by vibrations in experiments (Bolanowski et al., 1988; Strzalkowski, Ali & Bent, 2017). These are called the Pacinian (P, mediated by Pacini corpuscles) channel, the non-Pacinian I (NPI, mediated by Meissner corpuscles) channel, the non-Pacinian II (NPII, mediated by Ruffini end organs) channel, and the non-Pacini III (NPIII, mediated by Merkel cells) channel. For hairy skin, Bolanowski, Gescheider & Verrillo (1994) propose the presence of three channels.

Hence, the occurrence of spatial summation is controversial when applying lower frequencies (e.g., 25 or 40 Hz). While Verrillo (1966a, 1966b) could not demonstrate its presence in glabrous and hairy skin, it was Muijser (1990) who concluded a different outcome considering glabrous skin. For high-frequency vibratory stimuli, detected by Pacini corpuscles with an optimal sensitivity range of 200–400 Hz (Wilska, 1954), the results are more obvious. Spatial summation generally results in lower VPTs (increased sensitivity) as the size of the contactor area increases (Gescheider et al., 1999). This may result in a clearer perception of the vibratory stimuli following spatial summation. Hence, the question arises whether this may also lead to a reduced variability of the data potentially resulting in an improved capability of statistical tests to detect differences. However, no studies have investigated this issue yet, and many clinical studies use a single and rather small contactor instead of a larger contactor size to assess VPTs, also in hairy skin.

To clarify the above-mentioned issues, the aim of the present study was to determine (a) whether high and low frequencies exhibit spatial summation measured at the hairy skin, and (b) whether a large contactor area (contactor matrix) is better able to identify differences of VPTs in hairy skin areas than a small contactor area (single contactor). The experiments were performed at three anatomical locations of the human torso, and at two frequencies (30 and 200 Hz). For each frequency, we hypothesized increased sensitivity for the matrix compared to the single contactor. Since cutaneous vibratory sensitivity is not equal within different torso locations (Wilska, 1954), we also hypothesized differences between the anatomical locations using the matrix and the single contactor. Additionally, we hypothesized a smaller variation of the VPTs when using the contactor matrix compared to the single contactor.

## Materials and Methods

Twenty healthy subjects with an even gender distribution took part in the experiments (median ± median absolute deviation: 23.0 ± 3.0 years, 171.0 ± 10.4 cm, 66.0 ± 11.9 kg). All subjects were free of neurological disorders which might influence VPTs. They were informed about the purpose of this study and gave their written informed consent to participate. This study was conducted according to the recommendations of the Declaration of Helsinki and was approved by the Ethics Committee of the Faculty of Behavioural and Social Sciences of the corresponding university (V-277-17-DS-KUS/WUS-22062018).

**Figure 1:**
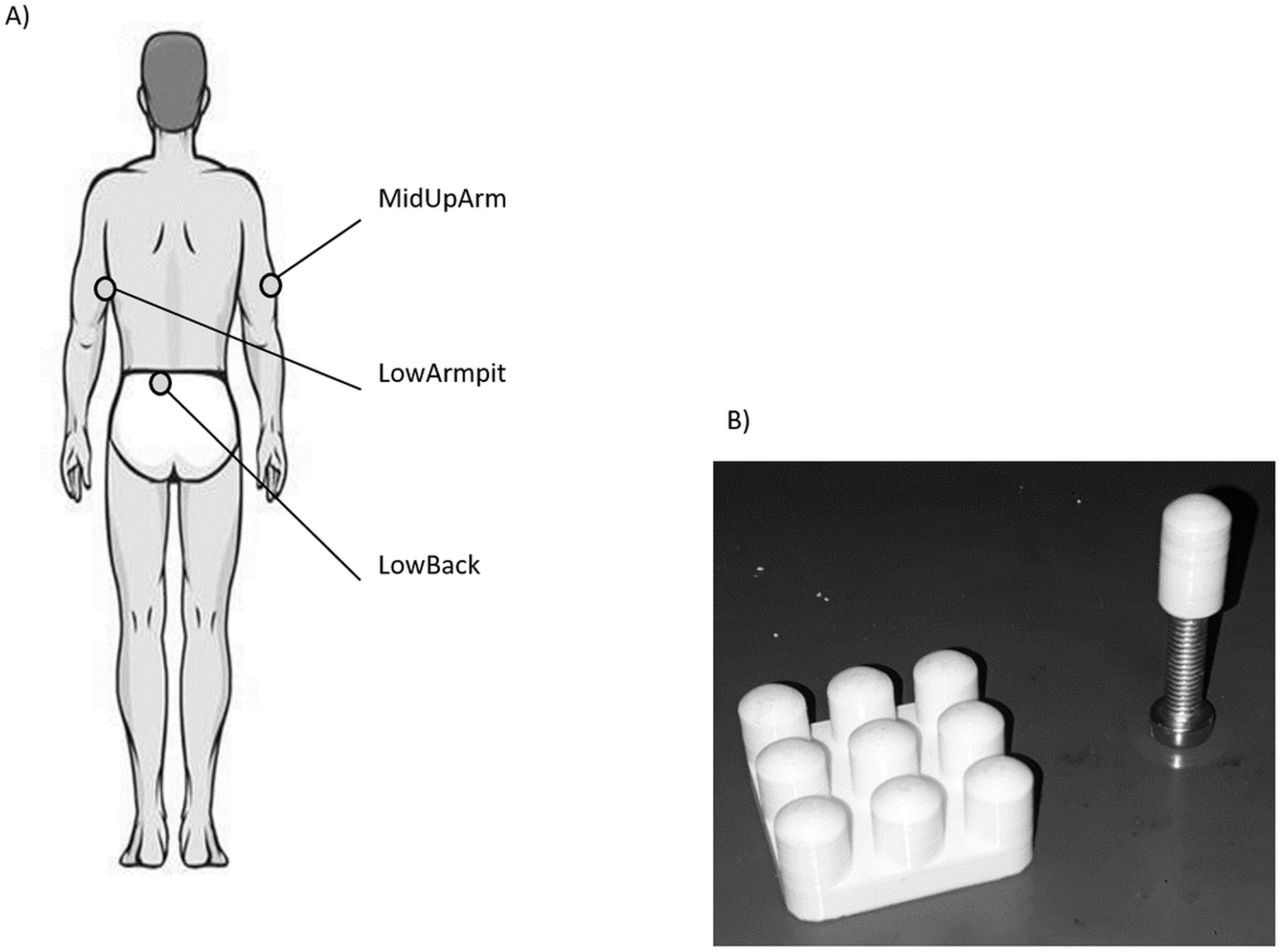
(A) Illustration of the three anatomical locations measured in this study. (B) Picture of the single contactor and contactor matrix used in this study.

After an acclimatization period of approx. 10 min, the three anatomical locations (Fig. 1A), namely the middle aspect of the lower back (LowBack), middle lateral aspect of the upper arm (MidUpArm), and the region just below the armpit (LowArmpit), were marked with a pen (hair was not removed). After a test trial, three VPTs were collected using a mini shaker (Type 4180; Brüel & Kjaer Vibro GmbH, Darmstadt, Germany) following a protocol similar to Mildren, Strzalkowski & Bent (2016). In short, a binary search program was used and participants had a hand held trigger to signalize the perception of a vibration stimulus. Vibration stimuli consisted of sinusoidal bursts with a duration of 2.0 s, with a randomized pause in between the bursts ranging between 2.0 and 5.0 s. The algorithm started with a supra-threshold stimulus and continuously decreased the stimulus amplitude by 50% of the preceding perceived stimulus. In case a stimulus was not perceived, the amplitude was set at the intermediate level between the last perceived stimulus and the unperceived stimulus. The VPTs were determined as the smallest sine amplitude perceived within the 11 iterations. One trial took about 60 s on average. The protocol also included catch stimuli (duration 4–7 s) to check whether participants pressed the trigger when there was no vibration stimulus. Such data was excluded from the experiments, which was, however, not the case.

For each location, VPTs were measured at 30 and 200 Hz using the single contactor (tip diameter: 7.8 mm (Nurse & Nigg, 1999), = 0.48 cm^2^, no surround, round-shaped edge, Fig. 1B), as well as the square contactor matrix (nine identical single contactors, 0.48 cm^2^ each) arranged in a 3 × 3 array resulting in a contactor surface of 9 × 0.48 cm^2^ = 4.32 cm^2^, no surround, inter-contactor distance: 1.0 cm, Fig. 1B. Anatomical locations, contactor conditions, and frequencies were randomized block-wise: The test trial and the subsequent three trials were always conducted together, for both frequencies and both contactor conditions. In addition, room (23.0 ± 2.0 °C, EN ISO/IEC 17025) and pre vs. post skin temperatures were monitored (LowBack: 32.6 ± 0.7 °C vs. 32.8 ± 1.3 °C; MiUpArm: 30.6 ± 1.8 °C vs. 31.7 ± 1.0 °C; LowArmpit: 32.3 ± 1.1 vs. 32.8 ± 0.7 °C, all median ± MAD). During the measurements, the same vertical force (controlled via a force transducer) and pressure were applied to each of the contactors, either in the single contactor or the matrix contactor condition (the angle was visually controlled and the longitudinal axis of the contactor was perpendicular with respect to the skin). This was important, since these features are known to influence VPTs (Cassella, Ashford & Kavanagh-Sharp, 2000). For the single contactor condition, the vertical force was set to 0.3 N. This corresponds to a pressure of 0.625 N/cm^2^. For the matrix, in order to ensure that each of its nine single contactors exerted the same vertical force on the skin, the overall vertical force was 0.3 N × 9 = 2.7 N. Hence, the same force and pressure was applied in both contactor conditions.

After the benefit of a logarithmization of median VPTs was proven, inferential statistical tests (pairwise Wilcoxon tests, Mann–Whitney-*U*-tests) were performed on log VPT data, see also Schmidt, Germano & Milani (2018). Due to the presence of three anatomical locations, the level of significance was corrected from α = 0.05 to α/3 = 0.017 when investigating differences between the locations.

## Results

**Figure 2:**
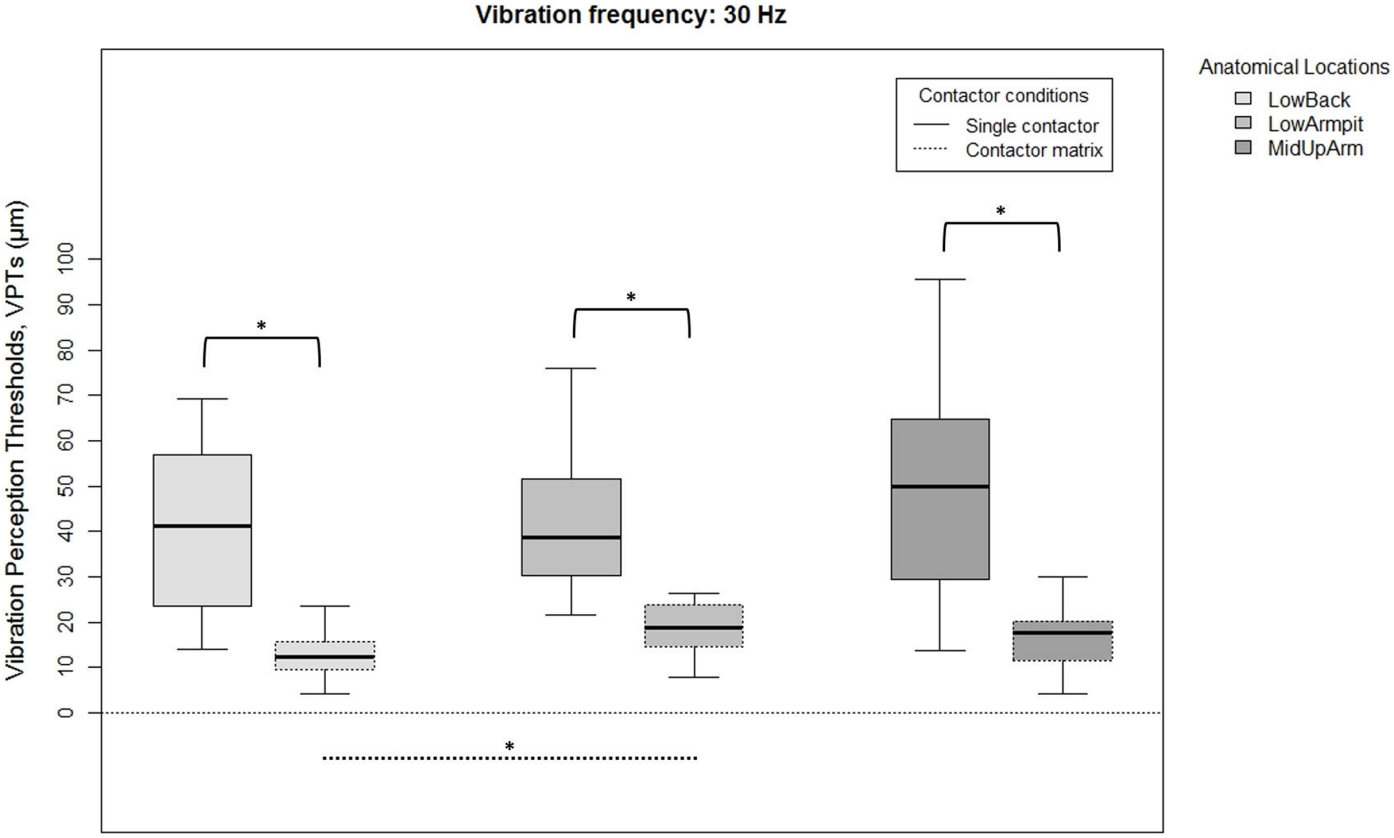
Raw vibration perception thresholds (VPTs) measured at 30 Hz. Raw vibration perception thresholds (VPTs) measured at 30 Hz highlighting significant differences between the single contactor vs. the contactor matrix (marked with square brackets), and between the three anatomical locations for each contactor condition (differences were evident only within the matrix condition, dotted lines). The horizontal line within each of the boxes represents the median, the boxes represent the interquartile range (IQR, 25th to 75th percentile). The lower and upper whiskers (25th percentile − (1.5 × IQR) and 75th percentile + (1.5 × IQR), resp.) do not contain outliers, which are not depicted in the boxplots. Inferential statistical tests were performed on logarithmized data. Significant differences: *p < 0.005, α = 0.017.

**Figure 3:**
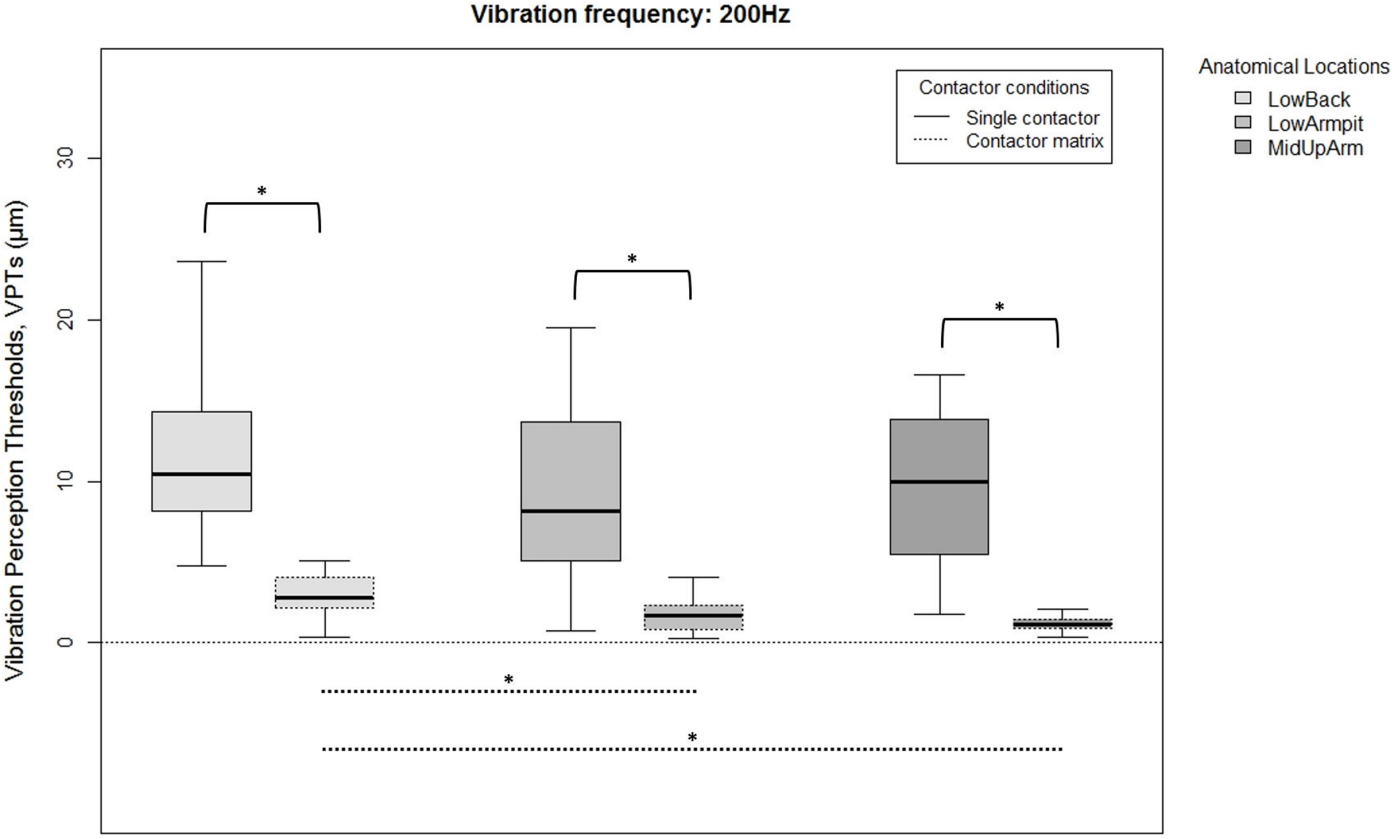
Raw vibration perception thresholds (VPTs) measured at 200 Hz. Raw vibration perception thresholds (VPTs) measured at 200 Hz highlighting significant differences between the single contactor vs. the contactor matrix (marked with square brackets), and between the three anatomical locations for each contactor condition (differences were evident only within the matrix condition, dotted lines). The horizontal line within each of the boxes represents the median, the boxes represent the interquartile range (IQR, 25th to 75th percentile). The lower and upper whiskers (25th percentile − (1.5 × IQR) and 75th percentile + (1.5 × IQR), resp.) do not contain outliers, which are not depicted in the boxplots. Inferential statistical tests were performed on logarithmized data. Significant differences: *p < 0.003, α = 0.017.

Since there were no overall gender differences for each location, frequency, or contactor condition, all 20 subjects were analyzed together. VPTs were significantly lower when using the contactor matrix compared to using the single contactor (Figs. 2 and 3). This was true for all three anatomical locations and both test frequencies (all *p* < 0.001).

When comparing differences between the three anatomical locations and for both frequencies, the single contactor did not identify significant differences (Figs. 2 and 3). When using the contactor matrix, three significant differences were found (Figs. 2 and 3): At 30 Hz, the LowBack was found to have significantly lower thresholds compared to LowArmpit (*p* = 0.004). At 200 Hz, significantly higher thresholds were present in LowBack compared to LowArmpit (*p* = 0.002) and MidUpArm (*p* < 0.001).

The variation within the three individual VPT trials (within subjects) comparing the single contactor vs. the contactor matrix is shown in Table 1. When using the matrix with 200 Hz, generally lower CoV (coefficient of variation) values were evident at the LowArmpit (75.0% of the subjects showed a lower CoV when using the matrix; mean CoV for single vs. matrix: 0.34 and 0.20, resp.) and MidUpArm (70.0%; mean CoV for single vs. matrix: 0.35 and 0.30, resp.). When using the contactor matrix with 30 Hz, only the Low Armpit showed an overall lower variability (55.0%; mean CoV for single vs. matrix: 0.29 and 0.26, resp.). We also calculated the CoV of the median VPTs over all twenty subjects (between subjects). When measuring with the contactor matrix, the LowBack at 200 Hz (single: 0.50, matrix: 0.46), LowBack at 30 Hz (single: 0.46, matrix: 0.41), and the LowArmpit at 30 Hz (single: 0.39, matrix: 0.30) exhibited lower values for the matrix compared to the single contactor.

**Table 1:** Percentages (%) of improvement in the coefficient of variation (CoV) at 30 and 200 Hz when using the contactor matrix. Each CoV calculation is based on the three individual VPTs (vibration perception threshold) trials for each subject. For example, 55.0% of the subjects had a lower CoV with the contactor matrix compared to the single contactor (30 Hz, LowArmpit).

CoV improvement with contactor matrix	LowArmpit	LowBack	MidUpArm
30 Hz (%)	55.0	30.0	27.8
200 Hz (%)	75.0	45.0	70.0

## Discussion

The present vibration thresholds of our work seem to fit with similar investigations. However, methodological differences (e.g., probe sizes or anatomical locations) have to be considered when doing comparisons. In terms of the few studies investigating hairy skin regions, our mean VPTs (single contactor of 0.48 cm^2^) were a bit higher compared to the VPTs Wilska (1954) found. This may be due to the fact that his contactor area was about twice as large (1.0 cm^2^). Considering different contactor areas when measuring the volar forearm, Verrillo (1966b) used sizes slightly different (0.32, 2.9 and 5.1 cm^2^) as in our study (0.48 and 4.32 cm^2^). Taking into account the differences in size and location, VPTs appear generally comparable to ours.

The finding that cutaneous vibratory sensitivity at high frequencies is higher when using large rather than small contactors is in line with previous investigations (Gescheider et al., 1999; Verrillo, 1992, 1971). However, we also found this behavior at low frequencies (30 Hz), which is in accordance with our hypothesis, but contrasts recent studies. In this regard, former studies confirmed that there is no spatial summation at low frequencies when investigating both glabrous (Mortimer, Zets & Cholewiak, 2007; Verrillo, 1966a) and hairy skin (Oliveira et al., 2016; Verrillo, 1966b). In contrast, Muijser (1990) noted much higher VPTs in other studies using a single contactor compared to his work using a large contactor matrix at glabrous skin. Also Bolanowski, Gescheider & Verrillo (1994) found that vibrotactile thresholds in the frequency range of about 30 Hz were lower (higher sensitivity) when using a large rather than a small contactor on hairy skin. They investigated the volar forearm using contactor sizes of 0.008 and 2.9 cm^2^.

To determine possible reasons for this discrepancy, knowledge of the underlying physiology of glabrous and hairy skin is essential. Hairy skin does not contain Meissner corpuscles (Munger & Ide, 1988), which are sensitive to low frequencies such as 30 Hz (Verrillo, 1977). In terms of such low frequency vibrations (30 Hz), it is currently not clear whether Vater–Pacini corpuscles are responsible for stimulus perception, because their optimal entrainment threshold is known to be in the upper frequency band at around 200 Hz (Strzalkowski, Ali & Bent, 2017). An earlier study revealed no perception of similarly low vibration frequencies stimulating the Pacinian system (Verrillo, 1992). Talbot et al. (1968) were unable to measure activity in the P channel at frequencies below 40 Hz when measuring the glabrous skin of monkey hands. However, they did not measure at 30 Hz. In contrast, more recent studies reveal a very different outcome. For example, in a microneurographic study (glabrous skin of the plantar foot), Strzalkowski, Ali & Bent (2017) showed that responses were evoked in FAII afferents even at low frequencies (30 Hz and lower). Additionally, Morioka & Griffin (2005) assumed that Vater–Pacini corpuscles might be involved at frequencies larger than 16 Hz. Finally, Birznieks et al. (2019) found that the P channel is capable of causing a conscious perception following vibrations at a frequency as low as 6 Hz (fingertips). Although these studies only consider glabrous skin, we conclude that a similar behavior of these receptors/channels applies in hairy skin. However, one should also consider the following: In these studies mentioned above, either substantially larger stimulus amplitudes were necessary to induce afferent firing or a conscious perception, or glabrous skin was measured. Our study, however, deals with subjective (not microneurography-based) methods aiming to determine absolute perception thresholds of the vibratory stimulus (hence, associated with much smaller amplitudes). In addition to this argument, there is an afferent channel known to be highly sensitive mediating low frequencies (e.g., 30 Hz) in hairy skin. This is accomplished by fibers associated with hair follicles and hair follicle afferents (Mahns et al., 2006; Verrillo, 1977). For vibratory frequencies of 4–45 Hz in hairy skin, Bolanowski, Gescheider & Verrillo (1994) identified the so called “NP _h mid_” channel (Non-Pacini channels in hairy skin at midrange frequencies): This channel is thought to be mediated by rapidly adapting fibers located quite superficially, with hair receptors as their sensory organs. Taking all of these arguments together, it is our primary assumption that the low frequency vibrations administered in our study (30 Hz) are mediated via the NP _h mid_ channel. However, an involvement of the P channel (associated with Vater–Pacini corpuscles) cannot be excluded.

It is currently unknown whether the NP _h mid_ channel (which we suppose is mainly responsive to our stimuli) is capable of spatial summation. Since we cannot entirely exclude a participation of the P channel, spatial summation seems one plausible explanation for our findings. In this case, our data would promote the assumption that the NP _h mid_ channel is capable of spatial summation, an assumption also made by Bolanowski, Gescheider & Verrillo (1994). As vibrations can propagate freely when not using a surround, spatial summation effects were shown to be particularly pronounced (Verrillo & Chamberlain, 1972), which seems to be present in our study. In another study performed by Morioka & Griffin (2005), the following effects were observed in glabrous skin: The likelihood to activate the P channel, especially at low frequencies, is increased when the stimulus area is larger and when vibrations are delivered without a surround. In terms of energy transfer and vibration propagation within the skin, a surround induces a gradient which makes it more likely to activate the NP I channel (which is not capable of spatial summation). Although the work of Morioka & Griffin (2005) was not performed on hairy skin, it further seems to support our explanation of spatial summation. In this regard, Gescheider et al. (2002) identified two possible mechanisms underlying spatial summation: The integration of neural activity and -foremost- the probability summation. The latter means that a larger contactor surface will raise the likelihood of activating highly sensitive receptors and afferents. Since the sensitivity of a sensory system is variable over a certain area, the spatial summation effect is less pronounced where a high density of highly sensitive receptors is present; or where a very low density is present (in this case, very few receptors and afferents will be stimulated using small or larger contactors). In contrast, the effect of probability summation is larger when an intermediate receptor density is present. Assuming that the NP _h mid_ channel is “responsible” for transmitting our stimuli, mediated by hair follicles as the sensory organ, this effect seems plausible: The back contains about the two-fold average density of hair follicles compared to the calf, but still considerably less than the forehead (Otberg et al., 2004). Unfortunately, the authors are unaware of any study investigating the density of Vater–Pacini corpuscles in hairy skin. If the NP _h mid_ channel is not capable of spatial summation, it seems likely that the P channel is responsive to low-frequency vibrations at a low amplitude level (around the absolute threshold level). This would be an interesting complement to the study performed by Birznieks et al. (2019).

However, there is another, alternative explanation (other than spatial summation) for the improved sensitivity of hairy skin (30 Hz) as the contactor area increased. This is related to energy and mechanics aspects, similarly proposed by Bolanowski, Gescheider & Verrillo (1994). It is evident that the structure of the matrix (nine contactors) induces zones of varying vertical pressures, shear strains, and stresses onto the skin. These would be greater compared to the single contactor conditions, possibly providing additional afferent information. In such a case, the VPTs would probably not be lowered due to a spatial summation effect. It has already been proven that these mechanical and stress-related properties actually influence afferent response profiles (Phillips & Johnson, 1981). However, this was related to glabrous skin, to other kinds of stimuli, and other afferent channels. Furthermore, in a pilot study, we compared VPTs obtained with the contactor matrix as used in this study (nine individual contactors, 3 × 3 cm) vs. VPTs obtained using a single and plane surface (also 3 × 3 cm). Clearly, these two conditions exhibit considerable differences in terms of energy transfer and skin deformation. Nevertheless, we were unable to find differences in VPTs. Therefore, we judge this aspect as rather unlikely and propose spatial summation effects of the NP _h mid_ and/or P channel as the most likely explanation.

Note that in another pilot study we also investigated applying the same overall vertical force (0.3 and 2.7 N) on the single contactor and matrix contactor. Although we observed the same finding of lower VPTs when using the matrix, future studies could further investigate different contactors using the same overall vertical force.

Probably as a consequence of the possible spatial summation mentioned above, our study revealed another important finding. Contradicting our hypothesis, only the matrix contactor identified differences between the anatomical locations. Hence, the contactor matrix seemed to exhibit a consistently superior ability to detect differences between the anatomical locations. For both tested frequencies and all locations, the single contactor was not able to identify significant differences, whereas the contactor matrix identified three differences. For 30 Hz, the LowBack was more sensitive than the LowArmpit. For 200 Hz, however, LowBack was the least sensitive region. Although we did not test spatial resolution properties of these locations, the differences in absolute perception thresholds might be relevant when selecting areas to apply devices for pattern recognition.

Previous studies Jones, Lockyer & Piateski (2006), Stronks, Parker & Barnes (2016), and Wilska (1954) identified considerable sensory variations across the torso. VPT differences within the head were also identified (Myles & Kalb, 2009; Oliveira et al., 2016). In clinical studies, determining VPTs is used to assess the presence of for example, diabetic neuropathies (Garrow & Boulton, 2006) or to predict the development of neuropathic ulcers (Young et al., 1994). VPTs may exhibit quite a large inter- and/or intra-subject variation (Wilska, 1954), hence, an improved ability of VPTs to detect changes might cause positive effects in this context, too. The large stimulating area (contactor matrix) facilitates the perception of vibratory stimuli and it seems this induces a higher capability to detect changes compared to a small stimulating area (single contactor). Additionally, when considering the boxplots (Figs. 2 and 3), our data seem to show a lower inter-subject variation when measuring with the contactor matrix compared to the single contactor. To investigate this assumption, we have calculated the coefficients of variation (CoVs) comparing the matrix vs. single contactor conditions. The CoVs calculated from the individual three VPTs for each participant, as well as based on the median VPTs showed that the superior performance of the matrix could not always be demonstrated. Hence, the superiority of the matrix is not consistently present and should be investigated in future studies, especially with patient populations.

## Conclusions

This study aimed to clarify whether or not high and low frequencies exhibit improved skin sensitivity when increasing the contactor areas, and whether or not a large contactor area identifies differences of VPTs rather than a small contactor size. For both frequencies, we hypothesized both an increased sensitivity as well as an improved ability of the matrix to detect changes compared to the single contactor. We found that an improved skin sensitivity due to larger contactor areas is present not only at high (200 Hz), but also at low (30 Hz) frequencies when applying vibratory stimuli at various regions at the human torso (hairy skin). The most likely explanation for this seems to be based on spatial summation properties of the underlying NP _h mid_ and/or P channel. Also, a large contactor size corresponded to a higher sensitivity to detect possible differences between VPTs of the torso. Future studies may take this into consideration when assessing VPTs, especially in a clinical context. This may be relevant when the aim is to differentiate between healthy and diseased populations, or after certain interventions. Furthermore, this study may highlight some important findings when developing new devices for pattern recognition for disabled people, such as the blind.

## Supplemental Information

10.7717/peerj.8479/supp-1 Supplemental Information 1 Raw data.

## Additional Information and Declarations

### Data Availability

The following information was supplied regarding data availability: The raw data is available as a Supplemental File.
